# Population structure analysis of *Salmonella* serovar Muenchen to redefine geno-serotyping using genome indexing approaches

**DOI:** 10.3389/fmicb.2025.1681711

**Published:** 2026-02-10

**Authors:** Padmini Ramachandran, Kranti Konganti, Amanda M. Windsor, Christopher J. Grim, Abani K. Pradhan

**Affiliations:** 1 Human Foods Program, U.S. Food and Drug Administration, College Park, MD, United States; 2 Department of Nutrition and Food Science, University of Maryland, College Park, MD, United States; 3 Center for Food Safety and Security Systems, University of Maryland, College Park, MD, United States

**Keywords:** genome indexing, serotyping, *Salmonella enterica* subsp. enterica Muenchen, polyphyletic, SeqSero2

## Abstract

Accurate identification of *Salmonella* serovars for source attribution in foodborne illness outbreaks. Traditional serotyping, which relies on antigenic properties, continues to serve as gold standard; however, advances in whole-genome sequencing (WGS) have enabled to the development of in-silico serotyping tools such as SeqSero2 and *Salmonella In Silico* Typing Resource (SISTR). Genome-indexing methods, such as bettercallsal, integrate DNA sketching and genome proximity analysis, have emerged as a promising tool for improving serovar resolution. This study examines the performance of DNA sketching-based serotyping in conjunction with established in-silico methods, focusing especially on *Salmonella* Muenchen, a polyphyletic serovar that ranks among the top 20 serovars linked with human infections in the United States. In this study, SeqSero2 was employed for antigen-based serotyping, SISTR for core genome Multi-locus Sequence Typing (cgMLST)-based phylogenetic clustering, pangenome analysis using PIRATE for microevolutionary insights, and bettercallsal for genome-indexing-based serovar calls. The results demonstrate that bettercallsal, leveraging the National Centre for Biotechnology Information (NCBI) Pathogen Detection database, enhances serovar resolution by incorporating genome proximity calls. The integration of SeqSero2 with bettercallsal yields complementary insights, maintaining historical serotyping nomenclature while enhancing serovar classification. This dual-tool strategy improves the discrimination of genomically distinct but antigenically similar serovars, therefore addressing limitations of traditional and molecular serotyping. Overall, integrating genome indexing through DNA sketching with validated in-silico serotyping tools establishes a robust framework for pathogen characterization. In this study, the tool is specifically applied for *Salmonella* serovar characterization. This methodology enhances the source attribution accuracy in outbreak investigations and establishes a framework for updating serovar classification in the era of genomic epidemiology.

## Introduction

Precise identification of *Salmonella enterica* serovars from food matrices and environmental samples is essential for accurate source attribution in illness outbreaks. A complicating factor is that *S. enterica* is divided into six subspecies and more than 2,600 identified serovars, classified according to the White–Kauffmann–Le Minor Scheme (Grimont and Weill, 2007; Guibourdenche et al., 2010). Over 70 years, epidemiological investigations of *Salmonella* have traditionally relied on serotyping, in which isolates are assigned into serovars based on the determination of somatic O antigens and flagellin H antigens that react with specific antisera (Grimont and Weill, 2007). Most *S. enterica* serovars possess two alternately expressed H antigens, referred to as phases. The phase-1 and phase-2 flagellin proteins are encoded by the *fliC* and *fljB* genes, respectively. The phase switch is regulated by the invertase *hin* and the *fliC* repressor gene *fljA* (Barco et al., 2014). Accordingly, the overall antigenic formula consists of three antigens: the first position denotes the somatic O antigens, while the second and third positions represents the two different flagellin H antigens, with each antigen position separated by a colon, i.e., O:H1:H2 (Grimont and Weill, 2007). Various combinations of 46 O antigens and 85 H antigens have yielded approximately 1,500 serovars within *S. enterica* subspecies *enterica* and about 1,000 serovars across the remaining subspecies of *S. enterica* together with *S. bongori* (Grimont and Weill, 2007; Achtman et al., 2020). Somatic O antigens are commonly encoded by the *rfb* region on the chromosome, whereas the secondary O antigens that have been characterized are generally encoded by accessory genes, many of which are located on mobile genetic elements. H antigens are flagellated proteins that may consist of either single epitopes or multiple epitopes. Multi-epitope H anti gens are referred as H antigen complexes. Such complexes can share a common epitope, such as “1,” while differing in one or more secondary epitopes. Because identical antigenic types can occur across different taxonomic lineages, subspecies identification is often incorporated into serotype designations. Despite its complexicity, serovar classification based on antigenic formulae, remains the gold standard.

With the rise of next-generation sequencing (NGS), genome-based typing (geno-serotyping) tools have gained prominence as alternatives to traditional serotyping. Several *in silico* classification tools employing NGS data are available for *Salmonella* (Zhang et al., 2019; Zhang et al., 2015; Yoshida et al., 2016). Because the genetic determinants of the O and H antigens are well characterized, multiple tools have been developed to infer *Salmonella* serovars directly from O and H antigen sequences derived from whole-genome sequencing data. These includes SeqSero2 (Zhang et al., 2019), the *Salmonella In Silico* Typing Resource (SISTR) (SISTR, 2017), *in silico* serovar prediction based on multilocus sequence typing (MLST) (Guerrero-Araya et al., 2021), which correlates sequence types with serovars. Tools such as Metric Oriented Sequence Typer (MOST) (Tewolde et al., 2016) and SISTR implement the MLST-based method, while SISTR also incorporates phylogenetic clustering of core genome MLST (cgMLST) and *k-mer* reference searches (Ondov et al., 2016). Several studies have investigated the utility of lineage-specific gene markers for identifying polyphyletic serovars (Achtman et al., 2020; Ashton et al., 2016). Benchmarking analyses have assessed the performance of *in silico* serotyping tools, often conducted by tool developers on unique datasets (Uelze et al., 2020). For example, MOST was validated on 6,900 isolates from Public Health England (Tewolde et al., 2016), SeqSero2 on ∼2,300 isolates submitted to the National Antimicrobial Resistance Monitoring System (NARMS) at the U.S. Centers for Disease Control and Prevention (CDC) (Zhang et al., 2019), and SISTR on 42,400 isolates from public databases (SISTR, 2017; EnteroBase, 2025). Comparative evaluations of these tools, such as SeqSero2 and SISTR, reveal varied performance that frequently depends on sample sets and methods. SeqSero2 is widely adopted because it leverages whole-genome sequencing while preserving the historical context of serotyping through antigen profiles. One key limitation of relying solely on antigen profile-based serotyping is that it attempts to map genotypic data onto phenotypic categories, potentially overlooking important genomic variation beyond the classical serotype definitions. *Salmonella* typing is currently in transition, the White–Kauffman–Le Minor scheme has not been updated since 2014, with the addition of 60 new serovars and sometimes in an attempt to achieve the simplification of antigen formulae have resulted in the collapse of two distinct serovars into one (Deng et al., 2025). As serovar assignment increasingly incorporates genetic relatedness, there is a growing demand for frequent updates to the scheme that are accessible and well disseminated (Brenner et al., 2000). In this context, integrating genomic information provides a more robust and informative framework for accurate serovar classification (Banerji et al., 2020).

Advancements in bioinformatics, specifically in the development of DNA sketching-based algorithms, have revolutionized the process of handling large sequencing datasets, permitting efficient and accurate analysis with the potential for near “real-time” processing (Rowe, 2019). bettercallsal was recently developed to address the need to identify multiple *Salmonella* serovars within metagenomic samples (Konganti et al., 2023). This tool leverages DNA sketching algorithms (Ondov et al., 2016) is built upon the *Salmonella* isolate metadata made available by the NCBI Pathogen Detection (PD) project (Sayers et al., 2022), which currently hosts around 2 million pathogen genomes, including nearly 730,000 *Salmonella* genomes as of February 20, 2025. The NCBI PD platform plays a vital role in traceback investigations by gathering clinical pathogen genomes with environmental pathogen genomes. By combining the NCBI PD *Salmonella* database (which uses SeqSero2 to establish the computed_type call), bettercallsal improves serovar detection by providing genome proximity-based predictions. Although originally designed for the discovery of multiple *Salmonella* serovars in metagenomic datasets, bettercallsal can also be used for the analysis of whole-genome sequencing of isolates. This increases its utility by enabling serovar identification based on genome similarity rather than relying solely on geno-serotyping. The combination of DNA sketching and genome proximity analysis makes bettercallsal a powerful tool for both metagenomic analyses and isolate-level serovar characterization (Konganti et al., 2023). Whole-genome sequencing (WGS) of *Salmonella* isolates derived from mixed cultures can sometimes pass quality control yet yield false “mixed” serovar calls due to the presence of multiple serovar. This highlights the need for enhanced QC tools that can detect such mixtures and progress the reliability of downstream analyses.

This study evaluates whether DNA sketching-based serotype calls can be effectively integrated with a validated geno-serotyping tool such as SeqSero2. Advances in genome indexing and bioinformatics methods offer the opportunity to update the serovar calling scheme by incorporating genome proximity while retaining the historical context of serotyping nomenclature. Such an approach could help elucidate genomic lineages of confounding serovars, including novel sequence types (STs) that lack formal approval and polyphyletic serovars sharing the same antigen scheme but differ genomically. bettercallsal, built on the NCBI Pathogen Detection platform, leverages SeqSero2-derived information while adding genome-indexing capabilities, thereby enhancing the resolution to distinguish among serovars with substantial genomic differences and detecting subtle variations between closely related serovars.

Focusing on *Salmonella* Muenchen, a top 20 *Salmonella* serovar associated with human illness in the United States and known to be a polyphyletic serovar (a group of bacteria that share the same O and H antigens but come from multiple, genetically distinct ancestors; lineages indicating that polyphyletic serovars are usually derived from multiple independent ancestors) according to MLST-based phylogeny, this study compares in silico serotyping results from SeqSero2, cgMLST typing using SISTR, pangenome exploration with PIRATE, and genome indexing through bettercallsal. This study hypothesizes that combining DNA sketching-based serotyping via bettercallsal with SeqSero2 provides a robust framework for serovar characterization, maximizing information retrieval while preserving serovar definitions through antigen profiles. Such an integrated strategy is crucial for improving source attribution in foodborne outbreak investigations.

## Methods

### Serovar Muenchen dataset

The dataset used in this study included 1,963 *Salmonella* Muenchen (antigen formula = 6,8:d:1,2, serogroup – C2–C3) genomes. Short Read Sequence Archive Run (SRR) as fastq.gz (raw reads) were downloaded from the NCBI Pathogen Detection database;^1^ the paired-end raw reads that were downloaded from NCBI and analyzed in this study will be mentioned as SRR throughout this manuscript. The SRRs submitted to NCBI are usually sequenced with Illumina sequencing platform.

In addition to Muenchen, other sequences were downloaded and used in this study, bringing the total dataset number to 1,990 SRRs. Outgroup sequences (*n* = 27 SRRs) included in this study are listed in Table 1. Some of these outgroups (e.g., Manhattan, Bovismorbificans, and Herston) were chosen for inclusion as their antigen formula is similar to that of *S*. Muenchen whereas others (e.g., Umbadah, Valdosta, and Newport) were chosen because they contributed to conflicting calls between bettercallsal and SeqSero2 identified during preliminary analyses.

**Table 1:** The outgroups and other serovars used in this study.

Serovars included in this study other than Muenchen (8:d:1,2)	Antigen formula	Serogroup	Number of SRRs used for this study
Berta	9:f,g,t:-	D1	1
Bovismorbificans	8:r:1,5	C2-C3	1
Enteritidis	9:g,m:-	D1	2
Hartford	7:y:e,n,x	C1	1
Heidelberg	4:r:1,2	B	1
Herston	8:d:e,n,z15	C2-C3	1
Javiana	9:l,z28:1,5	D1	1
Manhattan	8:d:1,5	C2-C3	7
Muenster	10:e,h:1,5	E1	1
Newport	8:e,h:1,2	C2-C3	2
Reading	4:e,h:1,5	B	1
Typhimurium	4:i:1,2	B	1
Uganda	3,10:l,z13:1,5	E1	1
Umbadah	1,3,19:d:1,2	E4	3
Valdosta	8:a:1,2	C2-C3	3

### Serotype analysis

The 1990 SRRs (Muenchen and outgroups) were analyzed using bettercallsal (version 0.7.0)^2^ for serovar calling. This tool employs an assembly-free genome indexing approach to categorize serovars and simultaneously incorporates fastMLST (Guerrero-Araya et al., 2021; Tseeman, 2025), an assembly-based sequence type analysis, as part of its workflow. Additionally, bettercallsal performs on-the-fly (otf) genome assembly using the MEGAHIT assembler (Li et al., 2015) on *Salmonella* only reads as identified by KMA aligner (Clausen et al., 2018), and the assembled genomes are used as part of the workflow output. Designed to identify multiple serovars, bettercallsal generates outputs for each process in separate folders named after the process.

SeqSero 2.0. was applied to the 1990 SRRs for direct serotype prediction. SeqSero 2.0^3^ was also used on the megahit assembled genomes as part of the bettercallsal analysis pipeline, with its default setting (k-mer based mode), to generate serovar calls from the SRRs, including outgroups sharing similar antigen profile and some serovars from the same serogroup of Muenchen (C2-C3). NCBI PD produces computed type calls for every *Salmonella* SRR using SeqSero2. Both the SeqSero2 results generated in this study (by running the tool locally on the SRRs) and the computed serotype predictions provided by NCBI (also generated using SeqSero2) were considered for comparison in this analysis. SeqSero2s, an updated version of SeqSero2, is now available on GitHub. In addition to serotyping, the pipeline also performs sequence typing based on assemblies (Deng et al., 2025). This updated version is not used to generate computed type calls on NCBI PD, therefore, this study did not incorporate this tool in analyses.

SISTR command line tool v1.0.2^4^ was used on genome assemblies (generated as part of bettercallsal analysis tool using MEGAHIT) of the 1990 SRRs for cgMLST analysis. cgMLST profiles were downloaded from https://enterobase.warwick.ac.uk/schemes/*Salmonella*.cgMLSTv2/ and clustered through Grape Tree^5^ and viewed through microreact^6^.

### Selection of analysis tools for conflicting serotype calls

**Figure 1:**
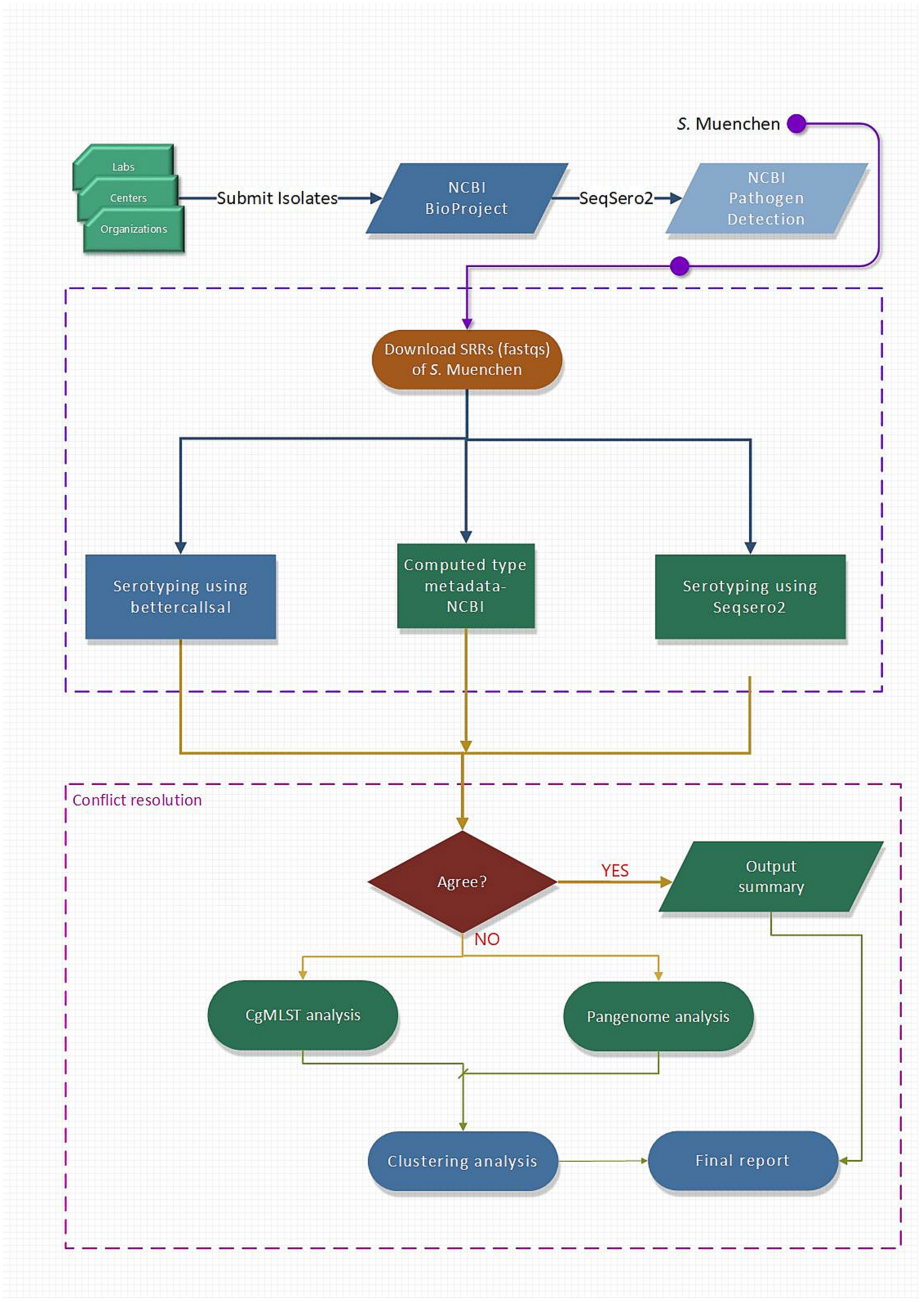
Schematic workflow used in this study. The workflow was designed for identifying and resolving serotyping discrepancies among *S.* Muenchen isolates in public databases. Isolate submissions by labs, centers, and organizations to NCBI Pathogen Detection (PD) are initially typed using SeqSero2. Sequence Read Archive Run (SRR) data for isolates labeled as *S.* Muenchen were downloaded, and serotype predictions were re-evaluated using bettercallsal, SeqSero2, and computed type metadata from NCBI. Discrepancies among these sources were flagged for conflict resolution. Discordant calls were further examined using core genome MLST (cgMLST), pangenome analysis, and clustering analysis to assess genomic divergence and determine serotype identity. A final report summarized findings for both concordant and discordant cases.

Serotype predictions made by computed type (from NCBI Pathogen Detection), SeqSero2 (using locally) and bettercallsal were compared and when these two tools disagreed, we incorporated several additional open-source tools into the workflow to resolve these conflicts (Figure 1). The tools selected for this study were chosen based on multiple criteria: they had to be open-source, capable of performing core genome assessments of *Salmonella* serovars, support rapid conflict resolution, and produce simple, interpretable outputs. Given that bettercallsal is a genome indexing tool, assembly-based sequence typing or serotyping approaches were deemed appropriate for resolving conflicts. The 7-gene Achtman Multi-Locus Sequence Typing (MLST) scheme, which profiles housekeeping genes from assemblies, is included as part of the bettercallsal analysis pipeline. The whole-genome MLST (wgMLST) tools were also evaluated, but were not to provide additional resolution compared to core-genome MLST (cgMLST). For cgMLST analysis, SISTR, which also incorporates hierarchical clustering to assess genome relatedness was used.

In addition to the typing approaches, pangenome analysis was executed to explore genome-wide variation as part of the conflict resolution process. Both Roary and PIRATE (Bayliss et al., 2019; Page et al., 2015) were evaluated as pangenome analysis tools, and PIRATE was ultimately selected for this study due to its ease of use and its ability to more effectively handle paralogous genes, which are common in bacterial genomes and can confound clustering. Furthermore, PIRATE’s approach to capturing accessory genome diversity and its robustness in generating gene presence/absence matrices made it particularly well suited for the comparative genomics required in this study.

### Phylogenetic analysis

Comparison and phylogenetic clustering of *fliC* alleles and *rfb* gene cluster was performed by RaXMLv.8.2.9 (Stamatakis, 2014).

### Pangenome analysis

MEGAHIT assembled genomes, generated as part of the bettercallsal analysis pipeline, were used for the pangenome (accessory plus core genome) analysis. Annotation and pangenome construction of these high-quality assemblies were performed with Prokka (v1.14.6) and PIRATE,^7^ sequentially (Bayliss et al., 2019; Seemann 2014). Genes identified in the pangenome were categorized into three different sets based on their prevalence across all strains analyzed: core genes were present in over 95% of isolates, shell genes were found between 15 and 95% isolates, while cloud genes were defined as those with a prevalence of less than 15%.

### Metadata

NCBI PD version PDG000000002.2727 was downloaded to obtain metadata information. The metadata fields that were relevant in this study include: computed type, isolation source, epi type, and SNP cluster (Supplementary Table 1).

## Results

### Serovar inference using bettercallsal compared to SeqSero2

Out of the 1,963 SRRs with NCBI PD computed type Muenchen, 140 SRRs showed conflicts between the genome indexing approach bettercallsal and computed type calls from NCBI. The serovar calls by bettercallsal other than Muenchen (antigen formula = 8:d:1,2, serogroup – C2–C3) included Valdosta (*n* = 90, antigen formula = 8:a:1,2, serogroup – C2–C3; *n* = 2 were part of dual serovar calls), Umbadah (*n* = 19, antigen formula = 1,3,19:d:1,2, serogroup – E4), no serovar calls with incomplete antigen formula (*n* = 32, I -:-:-), and Newport (*n* = 1, antigen formula = 8:e,h:1,2, serogroup – C2–C3 and one other SRRs called as Newport as part of dual serovar calls). 11 SRRs had multiple serovar calls (Tables 2, 3; Supplementary Table 1). The agreement between bettercallsal and computed type calls was 92.87%.

**Table 2:** Short Read Sequence Archive Run (SRRs) with multiple serovar calls identified by bettercallsal.

Input files	O group	fliC	fljB	SeqSero2.v1.3.1.	bcs_serotype1	bcs_serotype2	bcs_serotype3	ST	computed_types	Serovar overall	serovar_antigen	serovar_cgmlst	qc_status	serogroup
SRR5235480	3,10	d	1,2	Stormont	Muenchen	Anatum		112	Muenchen	Muenchen|Virginia|Manhattan|Yovokome	Muenchen|Virginia|Manhattan|Yovokome	Anatum	WARNING	C2-C3
SRR8661092	9	d	1,2	I 9	Muenchen	Enteritidis	I4…5…12.i…4.i…	83	Muenchen	Muenchen|Virginia|Manhattan|Yovokome	Muenchen|Virginia|Manhattan|Yovokome	Typhimurium	WARNING	C2-C3
SRR5740043	8	d	1,2	Muenchen	Muenchen	Heidelberg		83	Muenchen	Muenchen	Muenchen|Virginia|Manhattan|Yovokome	Muenchen	WARNING	C2-C3
SRR7292757	8	d	1,2	Muenchen	Muenchen	I4…5…12.i…4.i…		82	Muenchen	Muenchen|Virginia|Manhattan|Yovokome	Muenchen|Virginia|Manhattan|Yovokome	Typhimurium	WARNING	C2-C3
SRR1616752	8	d	1,2	Muenchen	Muenchen	Saintpaul		112	Muenchen	Muenchen	Muenchen|Virginia|Manhattan|Yovokome	Muenchen	WARNING	C2-C3
SRR25598226	8	i	1,2	Lindenburg	Muenchen	Typhimurium		83	Muenchen	Muenchen|Virginia|Manhattan|Yovokome	Muenchen|Virginia|Manhattan|Yovokome	Typhimurium	WARNING	C2-C3
SRR1973763	4	e, h	1,2	Saintpaul	Saintpaul	Uganda		684	Uganda	Saintpaul	Saintpaul|Reading	Saintpaul	WARNING	B
SRR5927258	4	d	1,2	Stanley	Umbadah	Coeln		112	Muenchen	Stanley|Eppendorf	Stanley|Eppendorf	Muenchen	WARNING	B
SRR5816801	7	d	1,2	Kisii	Umbadah	Newport		1,606	Muenchen	I C1:m,t:1,2	I C1:m,t:1,2	Muenchen	WARNING	C1
SRR5755460	9	g, m	1,2	I 9	Valdosta	Hillingdon		112	I-:-:-	I C2–C3:g,m:1,2	I C2-C3:g,m:1,2	Muenchen	WARNING	C2-C3
SRR5927267	9	l, z28	1,2	I 9	Valdosta	Javiana		4,162	Muenchen	Litchfield|Pakistan|Loanda|Fayed|Hiduddify	Litchfield|Pakistan|Loanda|Fayed|Hiduddify	Muenchen	WARNING	C2-C3

**Table 3:** Comparison of bettercallsal, SeqSero2, computed type, and cgMLST results for the 1963 SRRs.

Tool evaluated	SeqSero2 (run locally)	bcs_serotype1	serovar_cgmlst	computed_types
SeqSero2 (run locally)	1,963	1,933	1,854	1,857
bcs_serotype1	1,933	1,963	1,846	1,816
serovar_cgmlst	1,854	1,846	1,963	1,921
computed_types	1,857	1,816	1,921	1,963

The number of Short Read Sequence Archive Run (SRRs) that are in agreement between the tools are shown.

Out of 1,963 *Salmonella* Muenchen SRRs, 1,933 SRRs (98% agreement) showed concordant results between bettercallsal and SeqSero2 when run locally, both calling the serovar as Muenchen. However, 30 SRRs showed discrepancies between the two methods. Among these, 19 SRRs were identified as serovar Umbadah by bettercallsal, while both SeqSero2 and the NCBI PD call identified them as Muenchen. The remaining 11 SRRs included 7 with multiple serovar calls and 4 with other mismatches with incomplete antigen profiling, where the O antigen cannot be profiled by SeqSero2 (Table 3; Supplementary Table 1).

### Comparison between Seqsero2 and computed type calls

SeqSero2 when run locally on the MEGAHIT assembled genomes, has 106 SRR serovar calls that do not agree with computed type calls from NCBI Pathogen detection (Supplementary Table 1). Out of this 106, 90 Muenchen SRRs identified by computed type call were instead called Valdosta through SeqSero2 when run locally. Nine SRRs were called with incomplete antigen profile by SeqSero2 when run locally. Seven SRRs where called with a different serovar by SeqSero2, such as Stormont, Kisii, when run locally (Table 2).

### Core genome MLST using SISTR

Core genome MLST (cgMLST) analysis for all 1,990 SRRs was performed using SISTR. SISTR produces three primary serovar calls: overall, antigen, and cgMLST. The overall call is a consensus prediction derived from both antigen gene detection and cgMLST comparison. The antigen call is based solely on the presence of specific antigen-associated genes. The cgMLST call is determined by comparing the genome’s cgMLST profile to the SISTR database. This study focused exclusively on the cgMLST-derived serovar calls because cgMLST provides a high-resolution, allele-based classification that is not affected by missing or ambiguous antigen gene predictions. This approach ensures that serovar assignments are based on the underlying core genome variation rather than traditional antigenic schemes.

Examples of additional metrics output by SISTR of note to this study include QC status and serogroup (Supplementary Table 1). cgMLST overall calls agreed with bettercallsal except on the 19 SRRs that were called as Umbadah by bettercallsal. Those 19 SRRs were called Muenchen by SeqSero2, computed type and cgMLST.

### Multiple serovar calls

Out of 11 SRRs with multiple serovar calls identified by bettercallsal (Table 2), 6 had at least one call for serovar *Muenchen*, along with a secondary serovar such as *Anatum*, *Heidelberg*, or *Newport*. These 11 SRRs were analyzed using SeqSero2 (run locally), each produced a single serovar call (some other than Muenchen). However, cgMLST analysis for these 11 SRRs yielded quality check (QC) warnings. Among these, six SRRs had an overall cgMLST call of *Muenchen*, which corresponded with the *Muenchen* calls made by bettercallsal, even when a secondary serovar was also reported. The sequence types (STs) for the multiple serovar calls, are 112, 83, 1,606, 4,162 (fastMLST analysis as part of the bettercallsal analysis pipeline); all seem to indicate Muenchen.

### Cluster analysis

**Figure 2:**
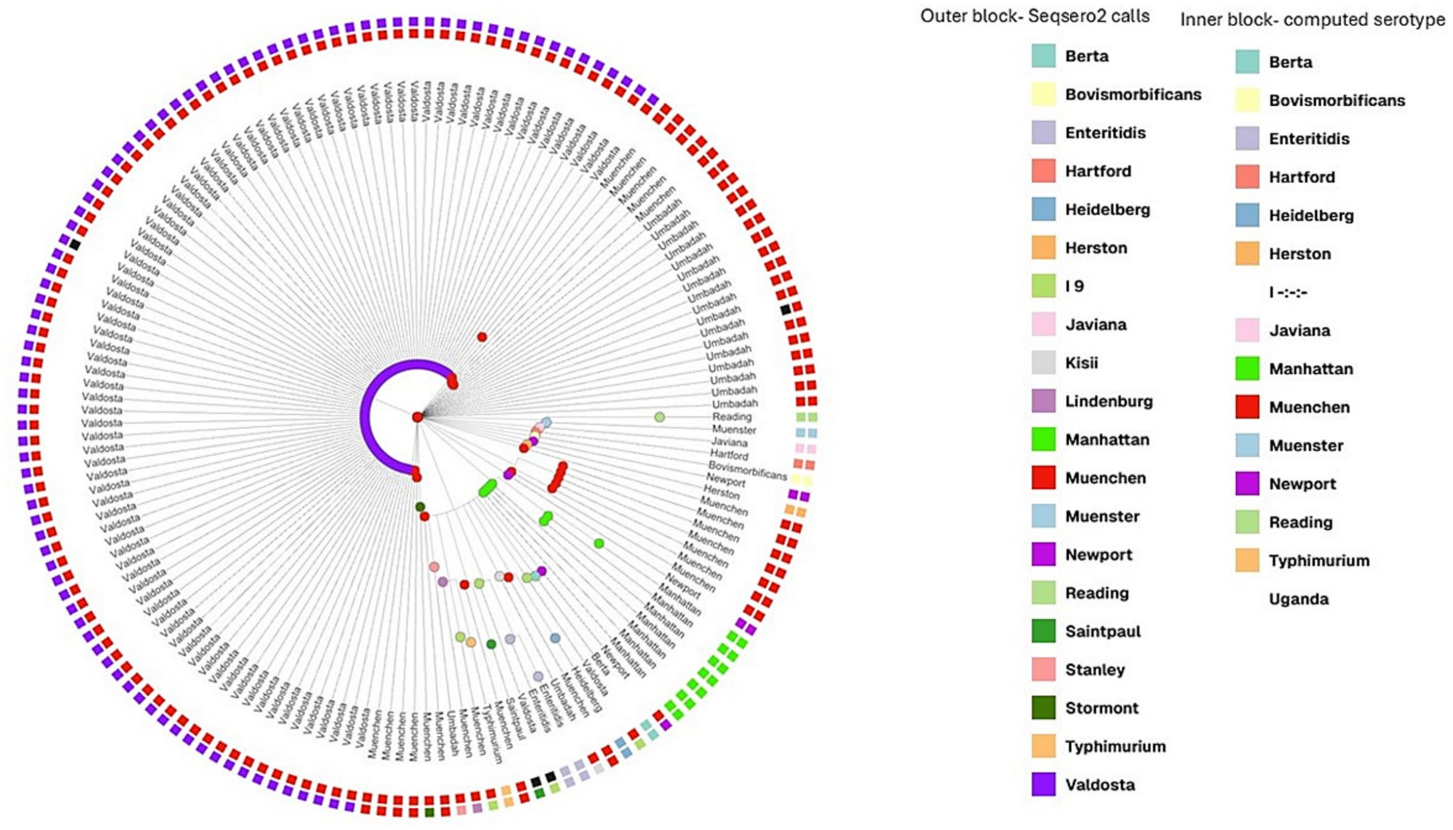
Grape tree hierarchical clustering of cgMLST allele profiles of the 140 SRRs that had conflicting calls between SeqSero2/computed type and bettercallsal along with the dual serovar calls and outgroups, with a total of 156 SRRs were used to create the hierarchical clustering. The node labels are calls from bettercallsal and the inner circle with the blocks are the computed type calls and the outer blocks are SeqSero2 calls when run locally.

A total of 156 SRR, including the 140 SRRs with serotype call conflicts, were included in cgMLST clustering analysis along with the 16 SRR with dual serovar calls and outgroups. GrapeTree was used for hierarchical clustering of the cgMLST alleles of these 156 SRRs. The tree was visualized using a web-based visualization called Microreact and serovar Typhimurium was used as the outgroup (Figure 2). The SRRs that were called Valdosta by cgMLST and bettercallsal clustered together and separately from Muenchen (Figure 2), whereas the SRRs that were called as Umbadah by bettercallsal, but as Muenchen by cgMLST and SeqSero2, formed a distinct clustered separate from both Muenchen and Valdosta (Figure 2). The SRRs with multiple serovar called by bettercallsal clustered closer to the outgroup (Figure 2).

Of the 156 SRRs included in cluster analysis, multiple serovars (2 or 3) were identified by bettercallsal in 11 SRRs (Table 2). In six of these, Muenchen was identified as one of the serovars; however, a secondary or tertiary serovar was also reported. The SISTR cgMLST pipeline confirmed Muenchen as the primary serovar in two of these six SRRs (Table 4). For conflicts between cgMLST and bettercallsal, 17 SRRs were identified as Umbadah by bettercallsal but were classified as Muenchen by cgMLST (Supplementary Table 1).

**Table 4:** Short Read Sequence Archive Run (SRRs) with a unique seven gene MLST profile without sequence types (ST) called.

ID	ST	aroC	dnaN	hemD	hisD	purE	sucA	thrA	O antigen call	fliC	fljB	SeqSero2 v1 3 1	bcs_serotype1	computed_types	serovar overall	serovar_antigen	serovar_cgmlst	cgmlst_ST	cgmlst_distance	cgmlst_found_loci	cgmlst_matching_alleles	qc_status	serogroup
SRR1384783	–	1,389?	–	–	–	–	814?	1,544?	8	d	1,2	Muenchen	Muenchen	Muenchen	Muenchen	Muenchen|Virginia|Manhattan|Yovokome	Muenchen	2,245,737,877	0.015151515	330	325	PASS	C2–C3
SRR15288878	–	41	9	21	12	483?	37	17	8	d	1,2	Muenchen	Muenchen	Muenchen	Muenchen	Muenchen|Virginia|Manhattan|Yovokome	Muenchen	119,922,267	0.027272727	330	321	PASS	C2–C3
SRR15334735	–	41	1,274?	43	1,827?	9	12	2	8	d	1,2	Muenchen	Muenchen	Muenchen	Muenchen	Muenchen|Virginia|Manhattan|Yovokome	Muenchen	18,888,484	0.018181818	330	324	PASS	C2–C3
SRR1604928	–	1,389?	42?	43	711	~9	1,254?	2	8	d	1,2	Muenchen	Muenchen	Muenchen	Muenchen	Muenchen|Virginia|Manhattan|Yovokome	Muenchen	3,854,783,697	0.012121212	330	326	PASS	C2–C3
SRR19276749	–	1,035?	42	43	58	1,147?	12	1,523?	8	d	1,2	Muenchen	Muenchen	Muenchen	Muenchen	Muenchen|Virginia|Manhattan|Yovokome	Muenchen	133,755,389	0.051515152	330	313	PASS	C2–C3
SRR23993264	–	1,376?	9	21	12	8	37	17	8	d	1,2	Muenchen	Muenchen	Muenchen	Muenchen	Muenchen|Virginia|Manhattan|Yovokome	Muenchen	119,922,267	0.027272727	330	321	PASS	C2–C3
SRR3049781	–	1,035?	9	21	12	8	37	17	8	d	1,2	Muenchen	Muenchen	Muenchen	Muenchen	Muenchen|Virginia|Manhattan|Yovokome	Muenchen	119,922,267	0.027272727	330	321	PASS	C2–C3
SRR3295610	–	41	9	21	12	8	~37	17	8	d	1,2	Muenchen	Muenchen	Muenchen	Muenchen	Muenchen|Virginia|Manhattan|Yovokome	Muenchen	1,651,767,759	0.027272727	330	321	PASS	C2–C3
SRR5902687	–	1,035?	~9	21	12	8	37	17	8	d	1,2	Muenchen	Muenchen	Muenchen	Muenchen	Muenchen|Virginia|Manhattan|Yovokome	Muenchen	119,922,267	0.027272727	330	321	PASS	C2–C3
SRR6411070	–	–	–	–	1,827?	1,353?	969?	1,184?	8	d	1,2	Muenchen	Muenchen	Muenchen	Muenchen	Muenchen|Virginia|Manhattan|Yovokome	Muenchen	3,601,647,004	0.024242424	330	322	PASS	C2–C3
SRR6685022	–	41	42	43	58	9	~12	2	8	d	1,2	Muenchen	Muenchen	Muenchen	Muenchen	Muenchen|Virginia|Manhattan|Yovokome	Muenchen	750,306,322	0.015151515	330	325	PASS	C2–C3
SRR7351409	–	1,035?	42	757?	58	9	12	1,548?	8	d	1,2	Muenchen	Muenchen	Muenchen	Muenchen	Muenchen|Virginia|Manhattan|Yovokome	Muenchen	1,127,678,313	0.033333333	330	319	PASS	C2–C3
SRR7358344	–	1,387?	42	43	58	9	12	2	8	d	1,2	Muenchen	Muenchen	Muenchen	Muenchen	Muenchen|Virginia|Manhattan|Yovokome	Muenchen	1,638,330,925	0.012121212	330	326	PASS	C2–C3
SRR7439592	–	41	42	43	1,827?	9	12	1,535?	8	d	1,2	Muenchen	Muenchen	Muenchen	Muenchen	Muenchen|Virginia|Manhattan|Yovokome	Muenchen	3,464,141,490	0.015151515	330	325	PASS	C2–C3
SRR7450842	–	1,389?	42	43	12	9	12	2	8	d	1,2	Muenchen	Muenchen	Muenchen	Muenchen	Muenchen|Virginia|Manhattan|Yovokome	Muenchen	18,888,484	0.018181818	330	324	PASS	C2–C3
SRR8201771	–	41	13	10	~12	5	19	12	8	d	1,2	Muenchen	Muenchen	Muenchen	Muenchen	Muenchen|Virginia|Manhattan|Yovokome	Muenchen	3,138,491,238	0.03030303	330	320	PASS	C2–C3
SRR6807857	–	~41	42	299	58	9	12	2	8	d	1,2	Muenchen	Umbadah	Muenchen	Muenchen	Muenchen|Virginia|Manhattan|Yovokome	Muenchen	555,240,777	0.042424242	330	316	PASS	C2–C3
SRR5460888	–	41	801?	43	58	9	12	2	8	a	1,2	Valdosta	Valdosta	Muenchen	Valdosta|Doncaster	Valdosta|Doncaster	Muenchen	2,947,593,669	0.033333333	330	319	PASS	C2–C3
SRR7911452	–	41	42	43	12?	9	12	2	8	a	1,2	Valdosta	Valdosta	Muenchen	Valdosta|Doncaster	Valdosta|Doncaster	Muenchen	2,947,593,669	0.033333333	330	319	PASS	C2–C3
SRR8738327	–	1,035?	42	43	58	9	12	2	8	a	1,2	Valdosta	Valdosta	Muenchen	Valdosta|Doncaster	Valdosta|Doncaster	Muenchen	2,947,593,669	0.033333333	330	319	PASS	C2–C3

### Sequence type using fastMLST

fastMLST is part of the bettercallsal analysis pipeline applied on the MEGAHIT assemblies of the SRRs to retrieve the ST from the updated PubMLST database (PubMLST, 2024). ST calls for Muenchen appear diverse, the majority (ratio) are associated with ST 82 and ST 83 (Supplementary Table 1). Unique MLST profiles were identified in 20 SRRs, resulting in no ST being called (Table 4).

### Somatic antigen clusters and flagella clusters

**Figure 3:**
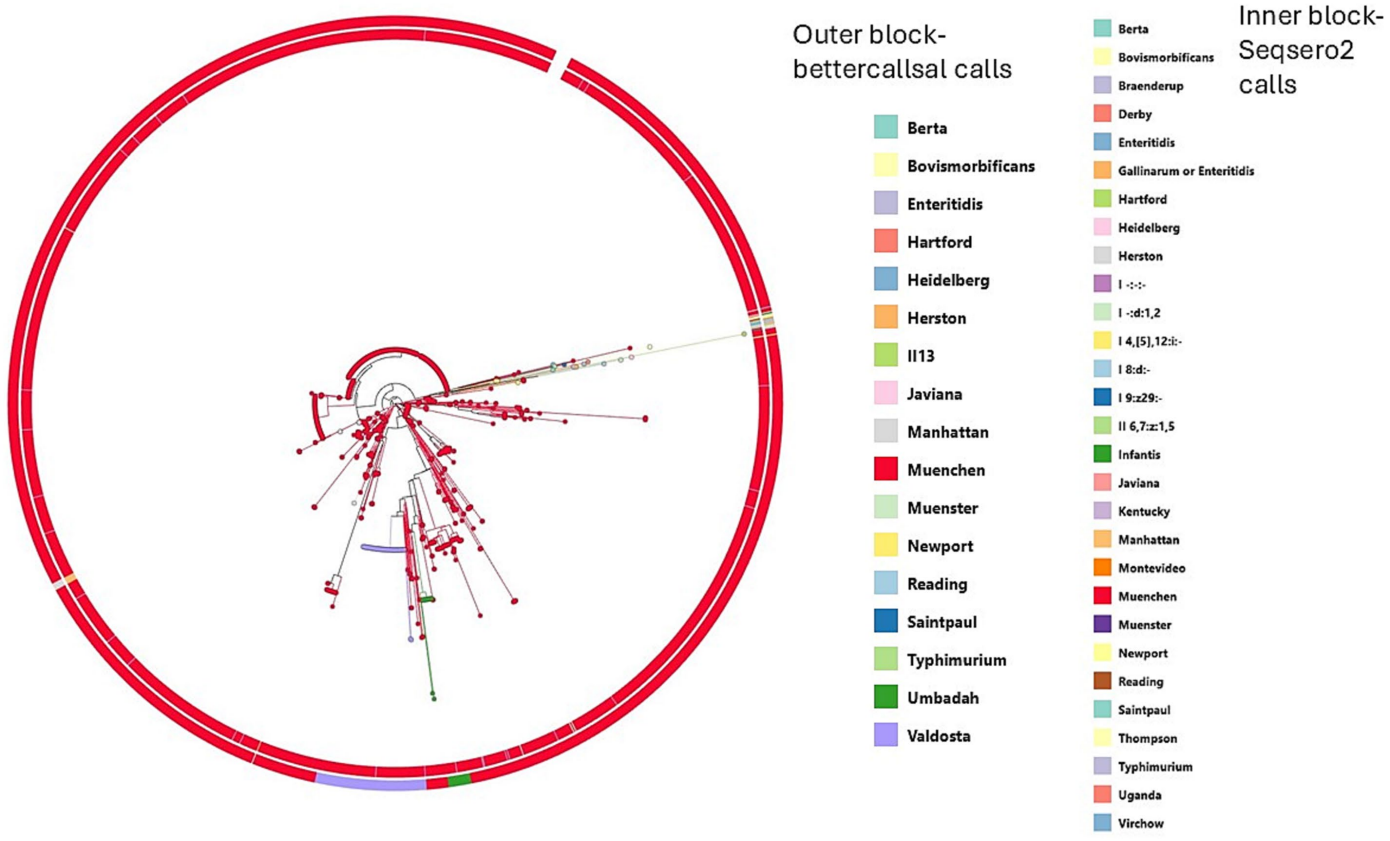
Maximum likelihood phylogeny based on *fliC* gene sequences from 155 *Salmonella Muenchen* isolates showing serotyping conflicts between computed type, SeqSero2, and bettercallsal. The tree was constructed using RAxML with 1,000 bootstrap replicates. Tip labels indicate the calls from bettercallsal, while outer and inner colored squares represent serovar predictions by SeqSero2 and computed type calls, respectively. Colors correspond to serovars as indicated in the legend.

From the Prokka-annotated genomes of the 1,990 SRRs, the 156 conflicting SRRs were explored more closely. *fliC* antigen sequences were extracted from the Prokka-annotated genomes. Alignment of *fliC* sequences using MUSCLE and the construction of a maximum likelihood tree revealed distinct clustering (Figure 3). SRRs identified as Valdosta by bettercallsal, with *fliC* profile changes from ‘d’ to ‘a’, clustered separately from typical Muenchen SRRs, which predominantly had a *fliC* antigen profile of ‘a’ (Figure 3). SRRs identified as Newport by bettercallsal, with *fliC* profile changes from ‘d’ to ‘e.h’, clustered separately from typical Muenchen.

**Figure 4:**
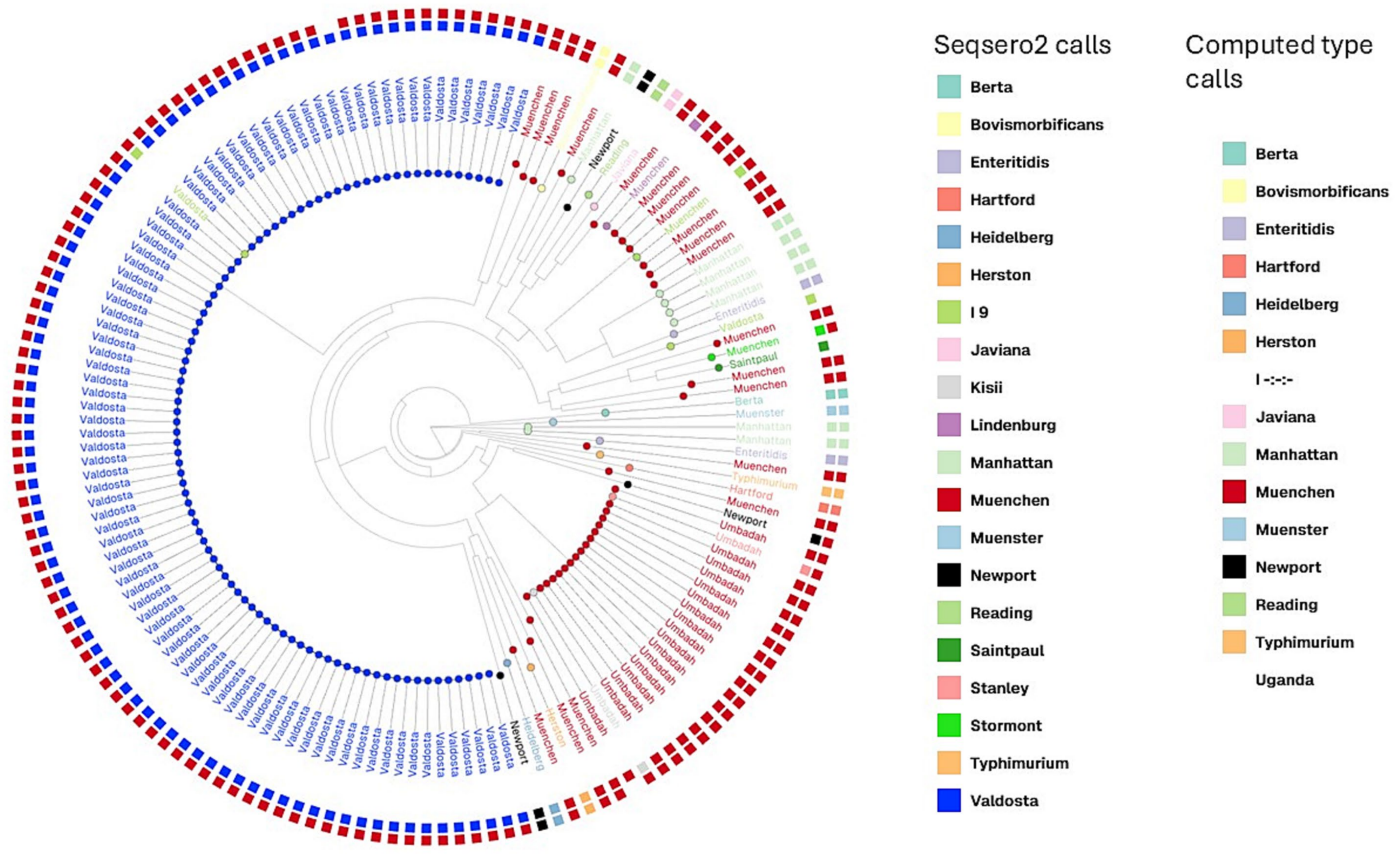
Maximum likelihood phylogeny based on *rfb* gene clusters from 155 *Salmonella Muenchen* isolates showing serotyping conflicts between computed type, SeqSero2 and bettercallsal. The tree was constructed using RAxML with 1,000 bootstrap replicates. Tip labels indicate the calls from bettercallsal, while outer and inner colored squares represent serovar predictions by SeqSero2 and computed type calls, respectively. Colors correspond to serovars as indicated in the legend.

Somatic O antigen sequences, for the 17 SRRs that were called as Umbadah (O antigen 1,3,19) by bettercallsal but called as Muenchen by all the other methods (cgMLST, SeqSero2 and computed type) were evaluated more closely. Alignment of *rfb* gene cluster sequences of the 19 SRRs, using MUSCLE and the construction of a maximum likelihood tree depicted no distinct clusters even though there were conflicting serovar calls. SRRs identified as Umbadah by bettercallsal, with O antigen profile changes from ‘8’ (serogroup C2-C3) to ‘1,3,19’ (serogroup E4), did not cluster separately from other Muenchen SRRs (Figure 4).

### Pangenome characteristics

From the Prokka-annotated genomes of the 1,940 Muenchen SRRs, pangenome analysis identified 3,159 core genes, 586 shell genes, and 8,139 cloud genes. Pangenome analysis of all the1,985 genome assemblies (Figure 4) and the resulting Newick tree revealed that sequences labeled as Valdosta (*n* = 90) and Umbadah (*n* = 19) by bettercallsal clustered separately from the remaining Muenchen (non-conflict) sequences. Particularly, these SRRs clustered with the respective true Valdosta (*n* = 3) and Umbadah (*n* = 3) sequences, supporting the genome-scale clustering. The 1,990 SRRs along with 3 SRRs with computed type Umbadah and 3 SRRs with computed type Valdosta were included in the pangenome analysis which revealed that the serovars that are called as Valdosta (*n* = 90) by bettercallsal were grouped into a separate branch along with the three true Valdosta SRRs (Figure 4).

## Discussion

This study reveals that an integrative approach to serotyping *Salmonella* using genome indexing with bettercallsal, geno-serotyping with SeqSero2, advances the robustness of serovar prediction and delivers new insights into microevolution within *Salmonella*.

Serovar designations are widely used for epidemiological purposes due to the assumption that they are discriminatory, represent a static subtyping scheme, and because serovars represent a globally understandable form of communication (Banerji et al., 2020). Various molecular methods have been proposed as alternative in this genomics era, like SeqSero2 based on White–Kauffman–LeMinor scheme, which generally works well but is less accurate for certain serogroups (Chattaway et al., 2021). For example, genes encoding antigenic epitopes can be imported by horizontal genetic exchange and homologous recombination from unrelated lineages. As a result, genetically related serovars could possess very different *fliC* or *fljB* alleles, whereas genetically distinct serovars can possess nearly identical alleles (Achtman et al., 2020; Smith et al., 1990). Thus, replacing serological determination with serotype-based molecular assays would maintain a system that does not necessarily reflect genetic relatedness. Although the genetic basis for many serotypes is well understood, it is unknown for some antigenic types. Geno-serotyping tools such as SeqSero2 and SISTR, which uses a combination of *in silico* serotyping and core gene multi-locus sequence typing (cgMLST), may incorrectly call an antigenic type when a close allele is not present in the database (Deng et al., 2025). Furthermore, some serotypes in the scheme are based on phenotypic characteristics other than O and H antigens, and inconsistencies in how variable epitopes are presented in the scheme make it difficult to assign some serotypes.

The genome indexing tool bettercallsal classifies serovars based on genetic relatedness rather than relying only on the sequences of O and H antigens. That said, bettercallsal, utilizes SeqSero2 by proxy since the computed type calls in the bettercallsal output are from NCBI PD (bettercallsal_db is underpinned by NCBI PD), which employs SeqSero2. SeqSero2 (when run locally) and bettercallsal agreed 98% of the time in this study. However, for novel STs and polyphyletic serovars, a comprehensive tool like bettercallsal offers deeper insights into genomic lineages by integrating assembly-based methods, sequence typing methods like fast MLST, cgMLST, and genome indexing approaches which helps in identifying the closest genomically related serovars. The genome indexing approach will give us genome proximity calls along with the calls that aligns with traditional serotyping. cgMLST clustering of the 90 SRRs that were called Valdosta by bettercallsal apart from the SRRs called Muenchen by bettercallsal, indicates that there is some genetic divergence among these SRRs.

Reports suggest the emergence of novel STs each year, consistent with the concept of an open *Salmonella* population (Banerji et al., 2020). To address this, the bettercallsal pipeline incorporates fastMLST on assemblies, which produces unique 7-gene MLST profiles for each sample (Guerrero-Araya et al., 2021). In this study, uncategorized MLST profiles were identified in 20 SRRS; subsequently, sequence type was not assigned (Table 4). These uncategorized MLST profiles may represent novel sequence types (STs). Among these 20 SRRs, bettercallsal identified the serotype as Valdosta for three SRRs and Umbadah for one SRR. Further investigation is needed to confirm whether these 19 SRRs without assigned ST represent novel alleles.

cgMLST typing using SISTR is highly informative, providing three types of outputs: Overall Serovar., Antigen-Based Serovar., and cgMLST-Based Serovar. Discrepancies were observed between antigen-based and cgMLST-based serovar predictions. Notably, SRRs identified as Valdosta by bettercallsal were consistently predicted as Valdosta by SISTR’s overall serovar results and SeqSero2 when run locally, which utilizes both antigen and cgMLST methods for serovar prediction. However, the cgMLST typing, which compares core genome and type against the SISTR database, indicated these genomes were Muenchen, not Valdosta. The Valdosta calls by bettercallsal are congruent with two of the cgMLST calls while the hierarchical clustering of the core genome proposes that these SRRs are genomically different from SRRs identified as Muenchen (Figure 2). Among the 11 SRRs that had multiple serovar calls by bettercallsal, cgMLST analysis clearly indicated that these SRRs did not pass quality control (Table 2). One SRR had three serovars called, while the remaining ten had dual serovar calls. SeqSero2, when run locally, assigned all 11 SRRs a single serovar. However, in cases of inter-serovar contamination, SeqSero2 can generate misleading results by combining the O antigen call from one serovar with the H1:H2 antigen call from another producing a hybrid antigen profile that matches an entirely different serovar (Table 2). For example, SRR5235480 was called as both Muenchen (8:d:1,2) and Anatum (3,10:e,h:1,6) by bettercallsal, but SeqSero2 classified it as Stormont (3,10:d:1,2), where the O antigen is from Anatum and the H antigens are from Muenchen. While the overall cgMLST call leaned toward Muenchen, the core genome cgMLST analysis supported Anatum, and the SRR ultimately failed cgMLST QC. The presence of multiple serovar calls is a strong indicator of potential inter-serovar contamination. This emphasizes the need for robust confirmatory tools like bettercallsal to detect and resolve complex typing conflicts.

There were 19 SRRs for which bettercallsal called the serovar Umbadah, but these same SRRs were called Muenchen by both SeqSero2 and cgMLST (Supplementary Table 1). Out of these 19 SRRs, two had more than one serovar called by bettercallsal and also did not pass QC for cgMLST analysis. There are only three SRRs of Valdosta and Umbadah each through computed type calls on NCBI Pathogen detection as of February 2025 and those SRRs were included as part of the analysis in this study. The hierarchical clustering of the core genome suggests that SRRs for which bettercallsal identified as Umbadah are genomically distinct from Muenchen (Figure 2).

One SRR (SRR8549089) was identified as *Salmonella* Muenchen (8:d:1,2) by computed type, but as *Salmonella* Newport (8:e,h:1,2) by SeqSero2 (run locally), bettercallsal, and cgMLST. To examine this discrepancy, two confirmed Newport SRRs were included in the outgroup set for comparison and clustering analysis. On NCBI Pathogen Detection, this conflicting SRR does not belong to any SNP cluster. The ST is 2,424 based on fastMLST analysis and this ST belongs to Newport. All evidence from these analysis supports its classification as Newport. This case highlights the importance of using a secondary tool to confirm SeqSero2-based computed type predictions, as discrepancies may occur. Although NCBI uses SeqSero2 to generate computed types, the specific implementation details are not publicly disclosed, there is a need for complementary tools like bettercallsal to identify and resolve such inconsistencies.

**Figure 5:**
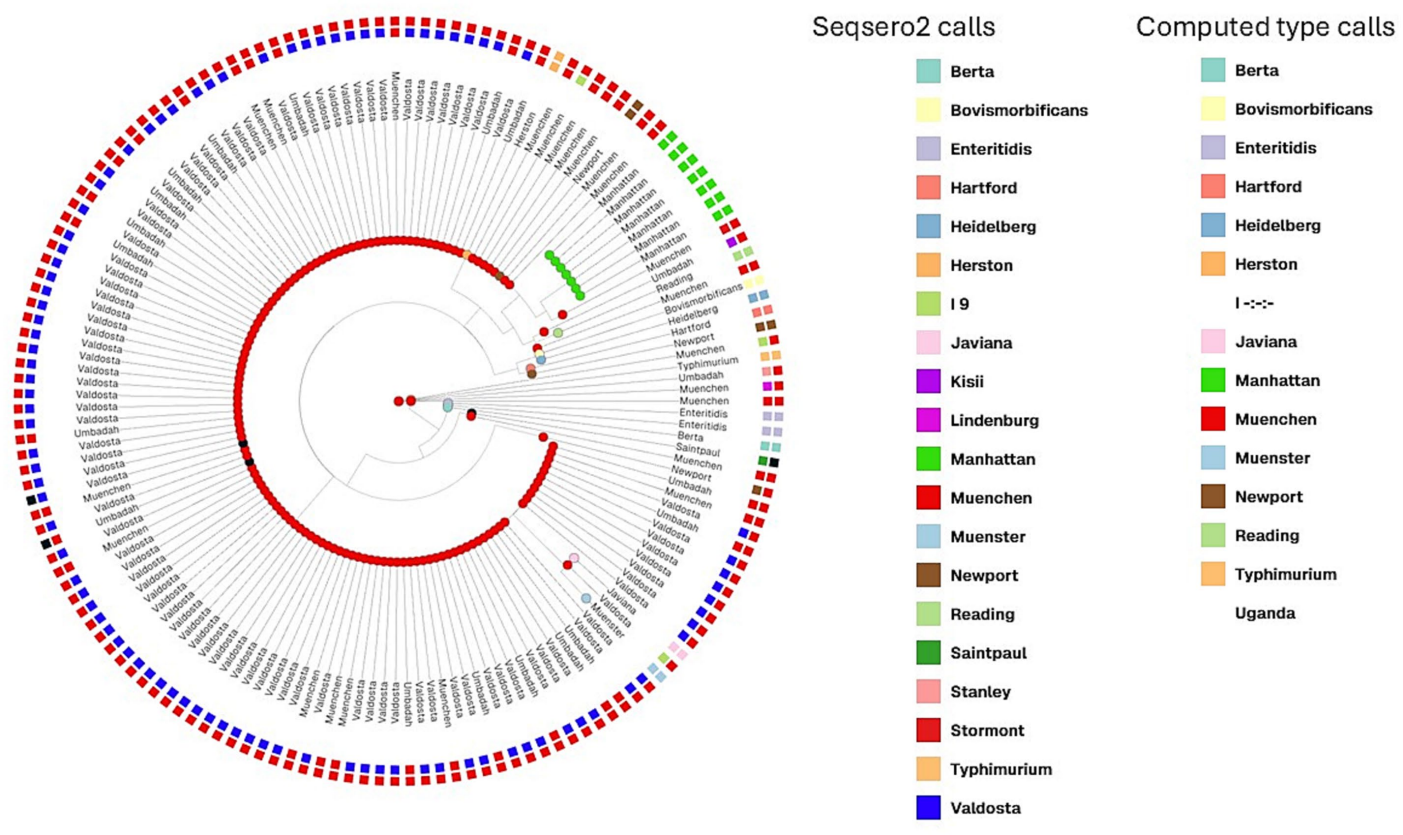
Pangenome analysis of 1990 SRRs of *S.* Muenchen and the outgroups. The node labels are calls from the bettercallsal and the blocks are the computed type calls by SeqSero2 (inner block) and bettercallsal calls (outer block). The outer purple blocks are called are Valdosta by bettercallsal and the dark green blocks are called as Umbadah by bettercallsal. Muenchen calls are marked in red.

Evaluating pangenomes has proven useful for enhanced surveillance, outbreak investigation, and microevolutionary exploration (Commichaux et al., 2023). The added public health value of pangenome data, however, depends on the unique genomic structure and microbial ecology of each *Salmonella* serotype and should be assessed within the context of serotype-specific population analyses (Cao et al., 2013). The population structure of *S. enterica* varies widely by serovar., with many serovars harboring two or more genetically divergent lineages. These patterns reflect differences in the relative contributions of recombination and mutation to genomic variation (Yang et al., 2025). Although over 1,500 *S. enterica* serovars have been defined based on surface antigen profiles, these serovars do not necessarily correspond to genetically uniform groups, as genes encoding surface antigens are frequently exchanged across lineages (Sangal et al., 2010). Subsequently, pangenome analyses and tools such as MLST must be applied to globally representative isolate collections to resolve population structures precisely (Cao et al., 2013). The SRRs that were called Umbadah (*n* = 19) by bettercallsal were also on a separate branch and clustered with the three SRRs with computed type Umbadah. However, these 19 SRRs were called Muenchen by cgMLST and SeqSero2. Somatic antigen alignment and clustering of the 19 SRRs did not delineate Umbadah SRRs into a separate cluster (Figure 4). But the pangenome analysis clustered these 19 SRRs separately from other Muenchen SRRs. (Figure 5). Pangenome analysis takes into consideration the entirety of the genomes and aims to incorporate the full range of genetic variation within a species. The effect of this is observed in the distinct clustering of the 90 Valdosta- and 19 Umbadah-assigned SRRs apart from the core Muenchen population (Figure 5). This finding establishes the value of serotype-specific pangenome investigations in refining surveillance and enhancing our understanding of microevolutionary patterns within *Salmonella*. Although traditional serotyping provides a valuable historical link to epidemiological data, higher-resolution subtyping is increasingly necessary to discriminate between lineages within *Salmonella* serovars. Tools like bettercallsal facilitate the identification of polyphyletic serovars by revealing distinct lineages. Recognizing these lineage-specific characteristics enhances outbreak detection and traceback investigations, highlighting the added value of genome-informed serotyping beyond conventional approaches.

SeqSero2, bettercallsal, and cgMLST reveal a 98% concordance rate in identifying *Salmonella enterica* serovar Muenchen. Each workflow offers distinct advantages for serovar prediction. SeqSero2 excels at detecting O, H1, and H2 antigen genes, assigning serotypes based on the White–Kauffmann–Le Minor scheme while leveraging whole-genome sequencing to maintain traditional nomenclature. However, challenges remain in understanding the genetic relatedness of certain serotypes, particularly among antigenically similar or monophasic variants. Recent enhancements to SeqSero2 that incorporate multilocus sequence typing (MLST) may help resolve some of these ambiguities. There are studies that have suggested other approaches such as Major Antigenic Clusters (MAC), MLST, and phylogeny-based typing have been proposed to move beyond antigen-based classification (Achtman et al., 2020; Chattaway et al., 2021). While these approaches offer promising directions, our study demonstrates that high-resolution, serotype-specific genomics analyses, within the context of existing nomenclature, continue to provide valuable insights into population structure and surveillance.

This study demonstrates the value of integrating multiple complementary methodologies for *Salmonella* serotype determination. The combination of these approaches can uncover novel sequence types (STs), dual serovar assignments, and genomic lineages of polyphyletic serovars, while ensuring both continuity with established frameworks and adaptability to emerging complexities in *Salmonella* genomics. Although this study focused on a single serovar., applying these integrated genomic approaches across all 2,600 *Salmonella* serovars holds the potential to transform our understanding of *Salmonella* population structure. Expanding this framework at scale could yield a more comprehensive, nuanced, and evolutionarily informed system of *Salmonella* typing, one that not only enhances genomic resolution but also strengthens global surveillance and public health response.

## Data Availability

The original contributions presented in the study are included in the article/Supplementary material, further inquiries can be directed to the corresponding author.

## Footnotes

1
https://www.ncbi.nlm.nih.gov/pathogens/

2
https://github.com/CFSAN-Biostatistics/bettercallsal

3
https://github.com/denglab/SeqSero2/

4
https://github.com/phac-nml/sistr_cmd

5
https://github.com/achtman-lab/GrapeTree

6
https://microreact.org/

7
https://github.com/SionBayliss/PIRATE

## Supplementary material

SUPPLEMENTARY TABLE 1 The Short Read Sequence Archive Run (SRR) used in this study along with the metadata and calls using Seqsero2, bettercallsal, cgMLST and computed type.
